# The diagnosis of hereditary angioedema with C1 inhibitor deficiency: a survey of Canadian physicians and laboratories

**DOI:** 10.1186/s13223-018-0307-0

**Published:** 2018-11-21

**Authors:** Xavier Charest-Morin, Stephen Betschel, Rozita Borici-Mazi, Amin Kanani, Gina Lacuesta, Georges-Étienne Rivard, Eric Wagner, Susan Wasserman, Bill Yang, Christian Drouet

**Affiliations:** 1 Department of Microbiology-Infectious Disease and Immunology, Laval University, Quebec, QC Canada; 2 Division of Clinical Immunology and Allergy, St. Michael’s Hospital, University of Toronto, Toronto, ON Canada; 3 Division of Allergy and Immunology, Department of Medicine, Queen’s University, Kingston, Canada; 4 Division of Allergy and Immunology, Department of Medicine, University of British Columbia, Vancouver, BC Canada; 5 Department of Medicine, Dalhousie University, Halifax, NS Canada; 6 Hematology/Oncology, Centre Hospitalier Universitaire Sainte-Justine, Montreal, QC Canada; 7 Immunology and Histocompatibility Laboratory, CHU de Quebec, Laval University, Quebec, QC Canada; 8 Department of Medicine, McMaster University, Hamilton, ON Canada; 9 University of Ottawa Medical School, Ottawa, ON Canada; 10 GREPI EA7408, University Grenoble Alpes, Grenoble, France; 11 Filière de santé Maladies Rares Immuno-Hématologiques (MaRIH), CHU Grenoble Alpes, Grenoble, France; 12 Present Address: INSERM U1016, Institut Cochin and Laboratoire d’Immunologie, Hôpital Cochin, AP-HP, Université Paris-Descartes, Paris, France

**Keywords:** Hereditary angioedema, Canada, Biological diagnostic, Diagnosis, C1-inhibitor

## Abstract

**Background:**

Hereditary angioedema due to C1 inhibitor deficiency (C1-INH-HAE) is an autosomal dominant disease resulting in random and unpredictable attacks of swelling. The swelling in C1-INH-HAE is a result of impaired regulation of bradykinin production. The fact that the array of tests needed to diagnose HAE is not always available to the treating physicians is challenging for them and their patients.

**Methods:**

The data for this article were extracted from two distinct surveys. The first survey was conducted among HAE treating physicians and aimed to determine the availability and utilization of the various assays performed to help the diagnosis of C1-INH-HAE. The second survey was conducted with the various laboratories across Canada that performs the assays used in the diagnosis of HAE. The aim of this survey was to determine the availability and profile of the various assays used in the diagnosis of C1-INH-HAE in Canada, thereby ultimately bringing a rational basis for the biological testing.

**Results:**

C1-INH functional assay was widely available in Canada (93%), but was only offered by a small numbers of hospitals meaning that there could be longer delays in the analysis of these samples that may explain why the physicians expressed a lower level of confidence in this assay (59%). Antigenic C1-INH was available to the vast majority of the physicians treating C1-INH-HAE (93%) and was considered reliable by 96% of the respondents. Antigenic C4 was found available to all Canadian physicians and, although with limited specificity, was considered very reliable by all the participants. This study revealed that 81% of physicians were able to order the antigenic C1q and the confidence in this assay was moderate (70%). Concerning genetic testing, the survey revealed that most of the CHAEN members never had to or couldn’t order this test.

**Conclusion:**

This study highlights the need for improved education and knowledge exchange, about biological assays available to Canadian physicians and their performance in proper diagnosis of C1-INH-HAE to improve confidence and access to relevant tests.

## Background

Hereditary angioedema (HAE) manifests itself by subcutaneous and submucous edemas mediated by bradykinin (BK), which is responsible for physiological vasopermeability [1–3]. HAE, like other BK related AEs, is characterized by recurrent episodes of oedema, lasting on average over 24 h that can affect the face, tongue, uvula, lips, upper and lower limbs and the genitourinary tract, which can seriously impact intimate relationships. The oedema can also involve the upper respiratory tract; there is therefore a major life-threatening risk in the event of laryngeal involvement. Abdominal attacks are very painful and are accompanied by nausea, vomiting and diarrhoea. Despite the chronic, debilitating and potentially life-threatening nature of the disease, HAE is often misdiagnosed. A European study has reported 8.5 years delay in diagnosis of HAE across eight European countries [4].

HAE associated with a C1-inhibitor deficiency (C1-INH-HAE) constitutes a complex syndrome, which causes a situation of functional gain of the contact phase with subsequent uncontrolled BK production. C1-INH is a serpin (serine protease inhibitor), which controls both activation and activity of many proteolytic systems, including contact phase representing the system that generates BK, also called Kallikrein–Kinin System (KKS) [5–7]. It consists of a group of 3 plasma proteins: factor XII (FXII, Hageman factor), prekallikrein (PK) and high-molecular-weight kininogen (HMWK) to which PK is combined (approx. 80%) [7]. In the absence of activation, the proteolytic system exclusively consists of inactive zymogens (proenzymes; < 0.15% active enzymes [8]). Cicardi et al. have proposed a classification which can distinguish in one part, the AEs due to C1-INH deficiency, of hereditary or acquired origin, and in another part, the AEs with normal C1-INH function, and an important group of unknown biological diagnosis [9].

Clinical diagnosis of HAE and other BK related AEs is based on the clinical description, the identification of triggers for the attacks, the response to medications during attacks, and possible familial history. In case of suspected HAE, the exploration of C1-INH remains the first-line test to distinguish between C1-INH-HAE from those with normal functioning C1-INH. This investigation includes the C1-INH serpin function, the antigenic C1-INH, and the distribution of C1-INH molecular species by plasma anti-C1-INH immunoblot. Obsolete recommendations have considered screening with antigenic C4 useful in an initial investigation of HAE, however this strategy has been found of low specificity because antigenic C4 is still normal in 27% (50/187) of individuals presenting with established C1-INH-HAE [10]. A familial diagnosis may be confirmed with genetic testing of *SERPING1* gene (OMIM 106100), with subsequent allele segregation of symptomatic and asymptomatic individuals. In most cases, the molecular abnormality affects allele expression, leading to a decrease in both antigenic C1-INH and C1-INH function (hereditary angioedema type I, HAE-I). In 15% of families, the variants affect allele product function, with a reduction of the plasma C1-INH function (HAE-II). A transient and partial decrease of C1-INH function can be observed in female individuals with AE under the effect of a trigger, such as the administration of estrogen [11]. In these situations, investigating *SERPING1* gene is not appropriate, but testing *F12*, *PLG*, *ANGPT1* or other genes is highly recommended.

Acquired angioedema due to C1-INH deficiency (C1-INH-AAE) can occur in conditions of acquired deficiencies of C1-INH that are not familial and inherited. A quantitative or functional C1-INH deficiency with negative family history, and sometimes low antigenic C1q and/or associated with anti-C1-INH antibodies of significant titres, contribute to a diagnosis of C1-INH-AAE. The screening for anti-C1-INH antibodies must be performed in patients above 50 years, with a recent appearance of attacks. C1-INH-AAE is caused (i) by a hyperactivation of the classical convertase combined with the presence of anti-C1-INH antibodies or (ii) secondary to a tumour proliferation, dysglobulinemia or an autoimmune disease, with a hypothesized proteolytic consumption of C1-INH; AAE symptoms can precede proliferation diagnoses by many years.

Despite well-detailed clinical and biological documentation, many BK related AEs remain to be classified [12]. Moreover, the array of tests required to confirm the diagnosis of HAE in the suspected cases is not always available to the practicing physician treating BK related AEs and HAE, because of a variety of reasons, thus posing challenges for them and their patients. The goal of our study was to survey practice patterns of Canadian physicians treating HAE patients and various laboratories conducting tests for the diagnosis of HAE.

## Methods

The data for this article were extracted from two distinct surveys carried out between May 2014 and November 2015. Missing or incomplete answers in both surveys were obtained through direct e-mails and phone calls. Both surveys were conducted by the Canadian Hereditary Angioedema Network (CHAEN), which is a non-profit organization that unites physicians committed to ensuring all HAE patients in Canada have access to excellent care that reflects current management and treatment guidelines, and works to promote research and education.

**Fig. 1:**
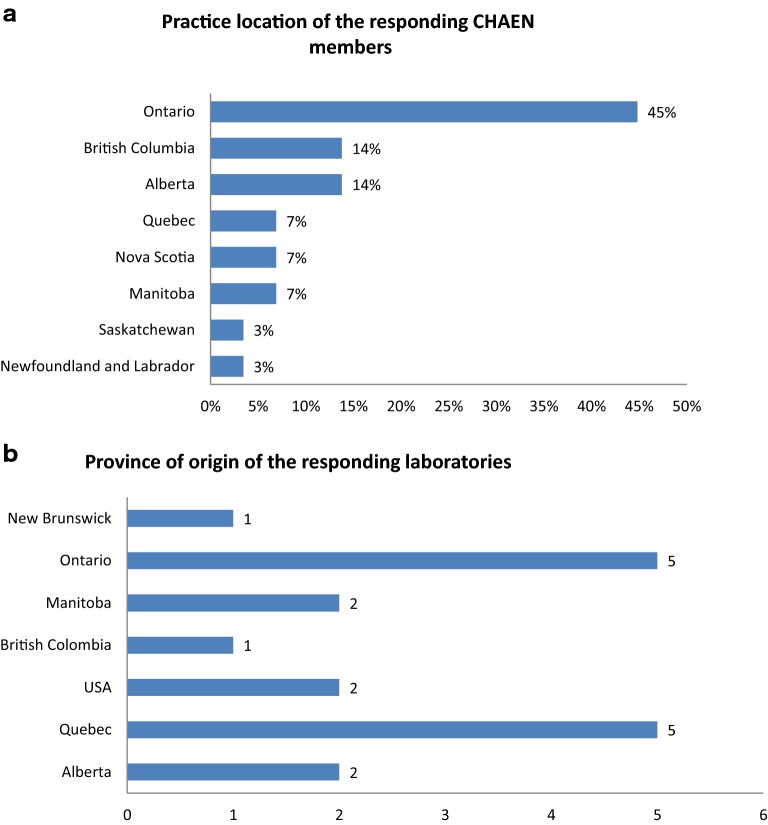
Practice location of the responding CHAEN members and province of origin of the laboratories that took either the MD survey or the Lab survey. CHAEN members who took the MD survey **a** or laboratories that took the Lab surveys **b** were distributed according to their province of origin to allow a better comparison between the results from both surveys

The first survey was conducted among CHAEN members. The aim of this survey was to determine the availability and utilization of the various assays usually performed to help in the diagnosis of C1-INH-HAE across Canada. Respondents were asked about the four main assays used in the diagnosis of C1-INH-HAE: C1-INH function using chromogenic assay based on the residual C1 s peptidase activity (Dade Behring GmbH, Liederbach Germany) or the ELISA test based on complex formation (Quidel Corp, San Diego CA USA), antigenic C1-INH, antigenic C4 and antigenic C1q. We also asked about genetic testing in C1-INH-HAE, which can confirm the diagnosis. Respondents were asked where the assays were performed, about the availability of the assays and how coverage was provided for the different assays. Finally, they were asked about their level of confidence in the various tests. Seventy-four percent of CHAEN members (29/39) were reached through this survey. The province of practice of the respondents is shown in Fig. 1a. Forty two percent of respondents practiced in Ontario.

The second survey was conducted with the various laboratories across Canada that performed one or many of the 4 assays listed above. Similarly, the aim of this survey was to determine the availability and profile of the various assays used in the diagnosis of C1-INH-HAE across Canada. The responding laboratories were inquired about the four main assays. The survey asked the following questions: which assays were used, the type of blood sample required to run the assays, the reference values for each assay and the costs associated with performing the assays. If the responding laboratories did not perform one or more of the assays, they were asked if the samples were referred to a different laboratory (private or public).

Replies to the survey were received from 16 of the 40 laboratories contacted across Canada and they are located in 6 provinces: Alberta (3), Ontario (5), Quebec (5), New Brunswick (1), Manitoba (2) and British Columbia (1; Fig. 1b). Information was also collected from three institutions outside the Canadian hospital networks where samples were sent by some physicians, one in Canada (In-Common Laboratories (ICL, North York, ON Canada), acting as a broker for hospital-based testing in Ontario) and two in the United States (National Jewish Health (Denver, CO USA) and Mayo Clinic (Rochester, MN USA)). Data were obtained from their respective websites or directly through e-mail contacts.

## Results

### C1-INH function

**Fig. 2:**
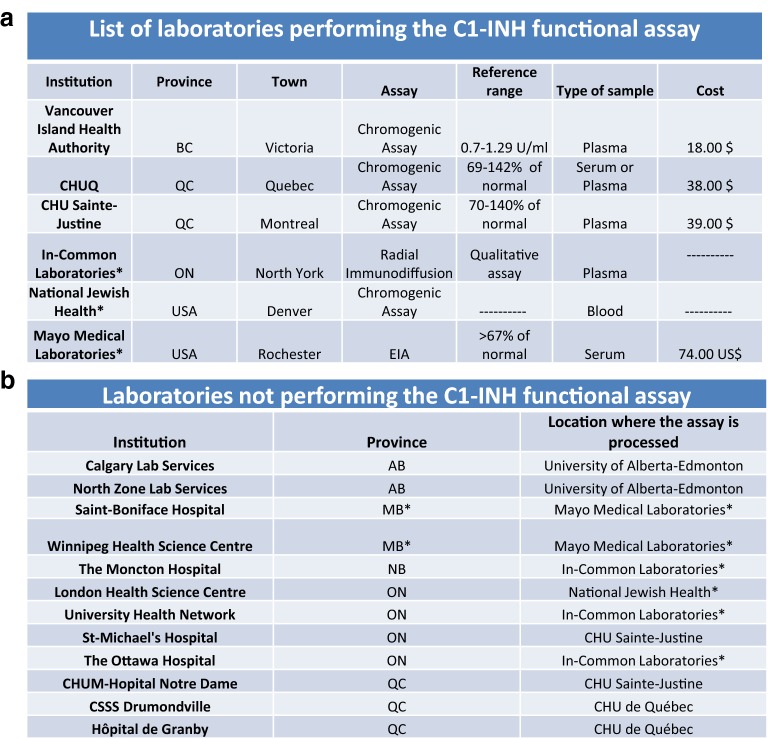
Availability of the C1-INH functional assay in Canada. **a** Laboratories that offered C1-INH functional testing at the time of the survey. When available, complementary information such as the type of assay, the reference range, the type of sample and the cost of this assay are available in this table. **b** List of laboratories that did not process locally the samples for the evaluation of the C1-INH function and location where this sample was processed

**Fig. 3:**
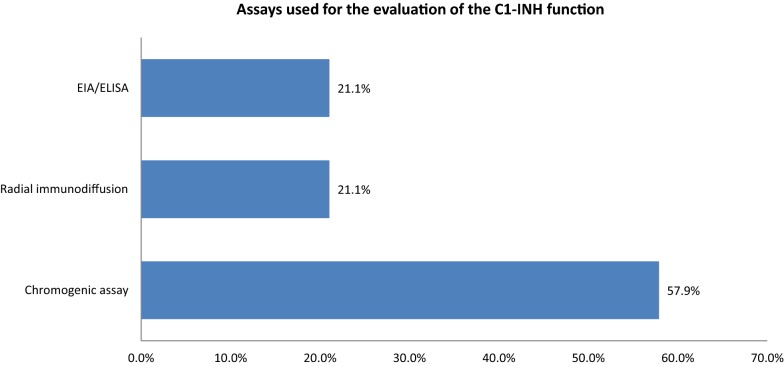
Use of C1-INH function assays by the CHAEN members. Data from both surveys were compiled and combined to determine frequency of use by Canadian physicians in the diagnosis of HAE

The C1-INH functional assay is commonly used when a physician suspects that a patient may have C1-INH-HAE. The CHAEN member’s survey showed that this assay is available to 93% of the responding CHAEN members. However, the laboratory survey showed that the assay is performed at very few locations (Fig. 2). According to results from both surveys, Manitoba and New-Brunswick do not offer this test and always send samples out to either other hospital-based or out of country laboratories (Fig. 2b). The CHU Sainte-Justine, CHU de Québec-Université Laval, University of Alberta Hospital, Vancouver Island Health Authority and the Vancouver General hospital were the investigative institutions (Fig. 2a). Ontario offered this test through In-Common Laboratories based at McMaster’s University. The most common assay used for the evaluation of C1-INH function was the chromogenic assay using C1s protease as target (Fig. 3). All the Canadian hospital-based laboratories performing this assay used a chromogenic assay, except for In-Common Laboratories at MacMaster’s University, which used a radial immunodiffusion assay when the responses from both surveys were collected. A chromogenic assay (National Jewish Health), or an ELISA assay (Mayo Clinic) were used in non-Canadian hospital-based laboratories. The reference ranges reported by the laboratories performing a chromogenic assay were all very uniform (min: 60–70% of normal; max: 140–145% of normal), in line with the use of a common commercial kit. As already reported [13], markedly disparate normal reference values were found when the complex ELISA has been used. The costs of the assays performed outside of the Canadian hospital systems were significantly higher than the cost of the chromogenic assay offered in non-Canadian laboratories. Provincial governments, in 93% of the cases, covered the cost of this assay. There was a surprisingly low level of confidence in this assay; with 41% of the respondents answering they did not trust the results. Many expressed concerns about the handling, storage and shipping of the samples. Also, they expressed concerns about the sensitivity and specificity of this assay. A new functional test using KKS proteases as targets has been developed with the highest specificity and the advantage of a linear dose–response in the readout [14]. Whatever the testing of C1-INH function used, the biological decision will be taken after retesting with an independent blood sample.

### Antigenic C1-INH

**Fig. 4:**
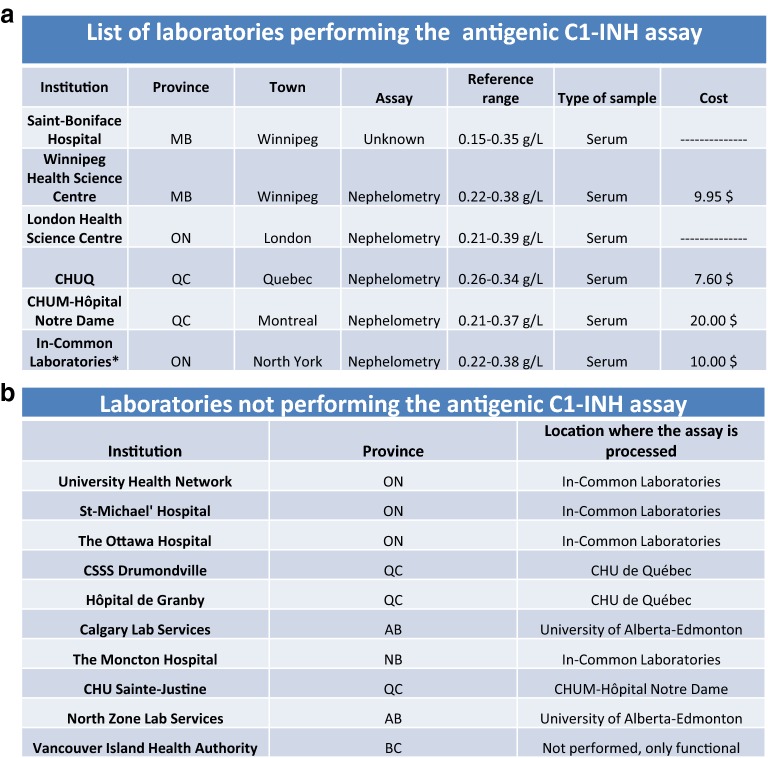
Availability of the antigenic C1-INH assay in Canada. **a** List of laboratories offering antigenic C1-INH testing at the time of the survey. When available, complementary information such as the type of assay, the reference values, the type of sample and the cost of this assay are available in this table. All samples were tested by nephelometry in relatively similar references ranges using serum samples. **b** List of laboratories that did not process locally the samples for the evaluation of antigenic C1-INH and location where this sample was processed

The CHAEN member’s survey demonstrated that 93% of the responding members were able to order the assay. This assay was performed in a large number of hospital-based laboratories across Canada. Hospitals in Alberta (University of Alberta Hospital), Manitoba (Saint-Boniface Hospital and Winnipeg Health Science Center), Ontario (London Health Science Center) and Quebec (CHU de Québec-Université Laval and CHUM) all offer the assay (Fig. 4a). However, many hospitals in Ontario and one hospital in New Brunswick subcontract their samples to In-Common Laboratories (Fig. 4b). In all Canadian laboratories, antigenic C1-INH is assayed by nephelometry on serum samples (Fig. 4). The reference ranges reported by the laboratories across Canada varied only slightly (min: 0.15–0.26 g/L; max: 0.34–0.39 g/L). However, some laboratories chose to only offer the functional assay and cancel the measurement of antigenic C1-INH in the cases where the functional assay yielded normal results. Contrasting with the low level of confidence in the C1-INH functional assay, there was a very high level of confidence in the antigenic C1-INH assay among the surveyed physicians. A total of 96% of the responding CHAEN members said they were confident in the results of this assay.

### Antigenic C4

The CHAEN member’s survey indicated that this assay is available to 100% of the responding CHAEN members. According to the data from the laboratory survey, all the samples were processed either on-site or at a laboratory within the local hospital network. Results from the laboratory survey suggested that the evaluation of antigenic C4 was done solely by nephelometry on serum samples. The reference ranges for this assay varied only slightly across Canada (min: 0.08–0.16 g/L; max: 0.30–0.65 g/L), suggesting the use of validated and standardized commercial reagents. The results from the CHAEN member’s survey showed that this assay was completely covered by the various provincial governments. The cost of this assay ranged from 4$ to 15$ which makes the measurement of antigenic C4 an affordable for HAE biological diagnostic. Physicians ordering the C4 antigenic level were 100% confident in the assay used to generate the results.

### Antigenic C1q

**Fig. 5:**
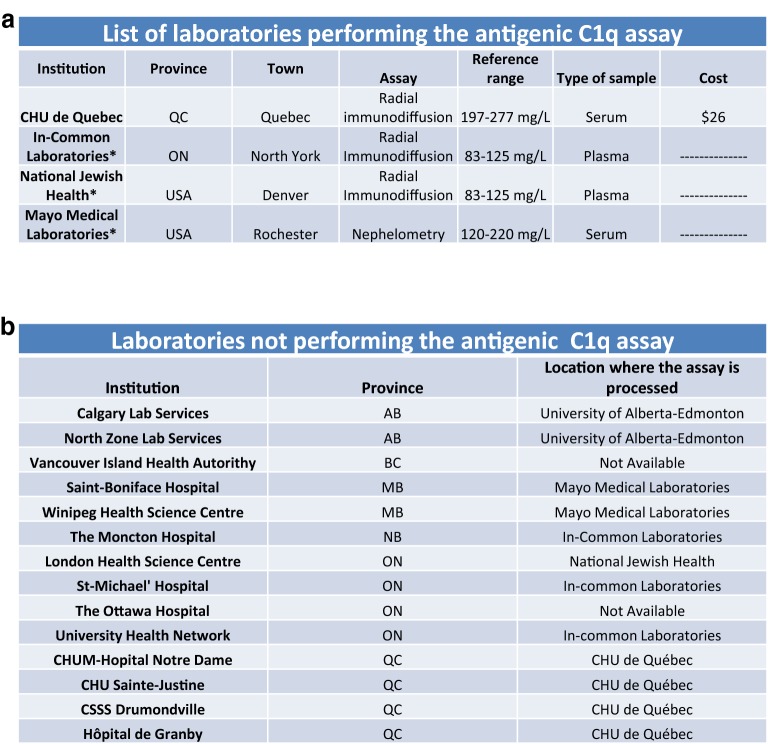
Availability of the antigenic C1q testing in Canada. **a** List of laboratories offering antigenic C1q at the time of the survey. When available, complementary information such as the type of assay, the reference range, the type of sample and the cost of this assay are available in this table. Samples collected by CHAEN members were processed either by radial immunodiffusion (70%) or nephelometry (30%). **b** List of laboratories that did not process locally the samples for the evaluation of the antigenic C1q and location where this sample was processed

The CHAEN member’s survey reported that 81% of the responding CHAEN members were able to order this assay. Still, this assay is significantly less available than the other assays considered in the survey. The vast majority of physicians who answered they could not order this assay mostly worked in private practice. The CHU de Québec-Université Laval was the only participating Canadian hospital laboratory that offered this assay (Fig. 5a). The In-Common Laboratories and the two US laboratories also offered this assay. The majority of antigenic C1q testing was performed in laboratories outside of the Canadian hospital-based system (Fig. 5b). The quantification of antigenic C1q is carried out either by radial immunodiffusion or by nephelometry (Fig. 5). The most common technique was the radial immunodiffusion. This technique is used in 3 out of 4 laboratories whereas nephelometry is used in one US laboratory. For the radial immunodiffusion assay, the reference values varied substantially between two laboratories (83–125 mg/L) and the Canadian laboratory (197–227 mg/L). This assay was covered in 80% of cases by the provincial governments. However, this assay may require special authorization before it can be sent to an out-of-province laboratory. When asked whether they were confident in the results of this assay, the responding CHAEN members had a moderate level of confidence in this assay with 52% being confident in the reported results while 24% were not. The remaining respondents could not answer this question because they never had to or could not order the test.

### Genetic testing

**Fig. 6:**
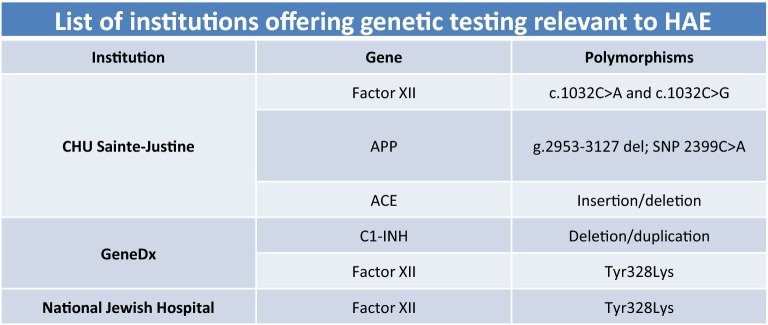
List of institutions offering genetic testing relevant to HAE. This table shows the different institutions providing genetic testing recommended in the diagnosis of HAE. The different variants investigated are also presented in this table

According to the answers provided by the CHAEN members, only 35% of them were able to order genetic testing for their patients. Testing for C1-INH gene (*SERPING1*) variants is not available in Canada and requests are being referred to some US laboratories, such as GeneDx (Baltimore MD USA). However, genetic testing covering genes encoding molecules involved in angioedema without C1-INH deficiency (FXII) or enzymes involved in kinin catabolism are available (Fig. 6). The CHU Sainte-Justine offers sequencing for the FXII gene (*F12*) and can detect two variants (c.1032C>A and c.1032C>G). The same center also offers sequencing for two kinin catabolism enzymes, aminopeptidase P (*XPNPEP2* gene), with g.2953–3127del and SNP c.2399C>A (dbSNP: rs3788853) and angiotensin-converting enzyme (*ACE* gene), with the insertion/deletion polymorphism (I/D, rs4646994). The GeneDx and the National Jewish Health laboratories also offer sequencing services for the *F12* gene. While being an expensive assay, genetic testing of FXII was significantly less expensive if performed at CHU Sainte-Justine. According to the respondents of the survey, genetic testing was covered in 88% of the cases but physicians generally must obtain governmental approval to have genetic testing performed out of province and covered. Not enough use of this service has been reported in the survey to draw any conclusion on the physicians’ level of confidence in this type of assays.

## Discussion

In this study, two distinct surveys were generated to investigate the use of assays in the diagnosis of C1-INH-HAE in Canada. The first survey was sent to CHAEN members i.e. physicians treating HAE patients, while the second survey was sent to Canadian laboratories performing assays developed in the diagnosis of C1-INH-HAE. Both these surveys revealed important information on the availability of the main assays as well as other crucial factors to consider when establishing a diagnosis such as confidence in the assays, reference ranges and the type of assays used. In the following lines, the assays are discussed in the order of their relevance for the disease.

C1-INH assays correspond to the disease target. In Canada, both antigenic C1-INH and function are available to the physicians in charge of treating HAE patients. Although the C1-INH functional assay is widely available to physicians, it is only offered by a small number of hospitals in Canada. The low number of facilities offering the C1-INH functional assay might imply that a longer storage period may be experienced before processing samples and running the assay. This may be a reason why almost half of the physicians surveyed expressed distrust in the results of this assay. Special care should always be given to the samples used for the measurement of the C1-INH function. It is important that the citrate or EDTA blood samples collected from the patients must be centrifuged within maximum 2 days of blood collection, then decanted and frozen at − 70 °C or below [15, 16]. A European-Canadian investigation stated that C1-INH function was stable in healthy individual samples both in whole blood and citrate plasma. In HAE patient whole blood, C1-INH was stable for up to 3 days in contrast with the shorter stability in isolated plasma [13]. Also, blood collection tubes must properly be filled to respect the optimal blood: anticoagulant ratio, thereby respecting the assay procedure and avoiding any dilution effect. This assay was performed mainly using a chromogenic assay outside of Ontario, although some laboratories used either an ELISA assay or a radial immunodiffusion assay. A published retrospective study of samples aimed at C1-INH function determination revealed that the use of the radial immunodiffusion assay may provide false negative results and that C1-INH-HAE or C1-INH-AAE may be misdiagnosed using radial immunodiffusion only [17, 18]. A comparative study performed in 2015 stated that both the ELISA and the chromogenic assay were accurate and efficient when the samples had been appropriately handled despite some discrepancies between the results from the two assays [19]. However, a previous study recommended the use of the chromogenic assay over the ELISA [13]. A new C1Inh function chromogenic assay has been developed providing an enzymatic readout, with the advantage of targeting all KKS proteases responsible for HK cleavage and BK production, as opposed to C1 s protease, in the chromogenic assay commonly used in clinical laboratories [14]. Another approach for C1-INH function was recently proposed in which the ability of this protein to form complexes with or to control either FXII or plasma kallikrein, [20]. The assays that used KKs as target are more relevant to C1-INH-HAE in line with the regulatory effect of C1-INH on BK generation via the KKS involved in angioedema attacks.

Identifying C1-INH species by immunoblot is a crucial step to differentiate between C1-INH-HAE-I and -II. HAE-I differs from HAE-II in that a pathological allele is not detectable in HAE-I whereas a defect in C1-INH function with expression of the wrong allele, i.e. without alteration in antigenic levels, is seen in HAE-II. Besides this type of analytical investigation is very useful to demonstrate a cleaved circulating C1-INH, which is a valuable contribution for diagnostic of AAE or a transient C1-INH cleavage in nlC1-INH-HAE. As mentioned, the C1-INH antigenic level assay was available to physicians in Canada and they had a very high level of confidence in this assay. Perhaps, the fact that this assay is mainly processed locally contributes to the higher confidence levels since this reduces the time between collection, sampling and laboratory analysis. Also, as opposed to the C1-INH functional assay, antigenic levels are less subject to sample degradation. The laboratory survey revealed that all the C1-INH antigenic level assays were performed using nephelometry, which is consistent with recent literature [21–23] whereas the radial immunodiffusion assay was more common in older literature [10, 24, 25]. More recently, an ELISA assay was also described to quantify the levels of C1-INH in citrated plasma [20].

These surveys revealed that measurement of antigenic C4 was widely available and considered reliable by physicians. However by its offside position considering the pathophysiological process, it is a real risk that this parameter will be relegated to a low priority [26]. Antigenic C4 was exclusively measured by nephelometry. Low C1-INH function leads to an uncontrolled activation of the classical complement pathway, with a subsequent reduction in circulating antigenic C4 [27]. It is important to keep in mind that a low antigenic C4 is only indicative of C1-INH-HAE, but it is not always conclusive because of the presence of C4AQo or C4BQo null alleles in healthy individuals, with allele frequency values in Anglo-Saxons of 0.169 and 0.185, respectively. Evidence suggests that normal antigenic C4 can be seen in patients with C1-INH-HAE [10, 26, 28]. However, measuring antigenic C4 during an HAE attack might improve sensitivity of the diagnostic in cases where normal antigenic C4 were noted in between attacks. While antigenic C4 has been considered as an initial step in supporting diagnosis, antigenic C1-INH and function must be measured even in the presence of normal antigenic C4 if C1-INH-HAE is suspected. According to a multicenter evaluation, the combined use of antigenic C4 and C1-INH function has 98% specificity for C1-INH-HAE with a 96% negative predictive value [10]. Recently it has been demonstrated that the kininogenase activity or kallikrein-like activity, a parameter associated with an uncontrolled KKS, displayed the highest sensitivity and sensibility for HAE diagnostic when associated to C1-INH function [29].

In C1-INH-HAE patients, antigenic C1q is normal, but in C1-INH-AAE, they are frequently low whenever anti-C1-INH antibody is identified [30]. The CHAEN member’s survey revealed that 81% of physicians were able to order the assay and the confidence in this assay was moderate. Being a disease with a significantly lower prevalence than C1-INH-HAE, it is normal for Canadian physicians to have less experience with this assay. This assay was processed at a very small number of facilities and only one of them was a Canadian hospital-based laboratory (CHU de Québec-Université Laval). In Canada, this assay is performed using radial immunodiffusion. One laboratory outside Canada reported using nephelometry, which is coherent with the literature [31, 32]. It has been demonstrated that both techniques offer the same sensitivity, but the results from nephelometry assay are obtained much faster since the radial immunodiffusion assay requires a longer incubation times [33]. There are also reported uses of ELISAs to determine antigenic C1q [34].

Genetic testing offers a complementary tool in the diagnosis of C1-INH-HAE or other forms of hereditary angioedema, especially in the case of nlC1-INH-HAE, with the knowledge of the segregation of the mutated allele(s) within family, including asymptomatic individuals. Genetic analysis of *SERPING1* gene is helpful to confirm familial diagnosis or, very rarely, when results from the classical assays are still inconclusive after using samples of poor quality. It is required for patients younger than 1-year of age, where C1-INH expression is still immature, compared to the adult. However, it is not available in Canada. Recently, Loules et al. reported a next-generation sequencing (NGS) platform that targets the entire *SERPING1* gene, offering a powerful approach for genetic analysis of patients with respect to C1-INH-HAE [35]. A real advantage given by NGS platform, when associated with copy number variation analysis, is to provide information about the size and localization of recombination fragments. Identification of family-specific variants is subsequently made possible by detecting the exact length and position of the deletion or insertion fragments after recombination between Alu repeated sequences. More than 500 *SERPING1* variants have been reported in the on-line Human Gene Mutation database HMGD^®^. Every new *SERPING1* variant needs to be validated for its association with decreased C1-INH function and clinical phenotype. De novo mutations within *SERPING1* gene are not uncommon, requiring further attention to family segregation. In proven C1-INH-HAE, genetic testing of APP and ACE deficiencies could potentially help in identifying patients with more frequent or more severe angioedema attacks [36, 37]. Variants within the *F12*, *PLG* and *ANGPT1* genes are described in nlC1-INH-HAE and are important to seek when C1-INH-HAE or C1-INH-AAE are ruled out. The CHAEN member’s survey revealed that most of the CHAEN members never had to or couldn’t order this test. Perhaps with the decreasing costs of genetic testing, this may become a much more common procedure in the future.

According to the CHAEN member’s survey, the main concerns expressed by physicians treating patients with HAE in Canada were: sample shipping and handling, having to wait a long time to get results, lack of recognized laboratories for genetic testing, diagnosis of nlC1-INH-HAE, testing of *F12* and other susceptibility genes (cost and availability) and ability to develop a reliable functional C1-INH assay, the basic testing for HAE diagnostic. The latter has since been addressed, at least for Ontario, as the In-Common Laboratories at MacMaster’s University now conduct measurement of C1-INH function via a chromogenic assay.

## Conclusion

The results of both surveys indicate the need for better education and information exchange amongst physicians treating HAE, about various assays available in Canada, their performance and relevance in proper diagnosis of HAE, creation of local laboratory expertise to eliminate sample processing issues and, overall, improve confidence and access to various tests. CHAEN could represent an important resource for its members to enhance collaboration and knowledge exchange amongst HAE consultants, laboratories and researchers within Canada and internationally. Such collaboration and knowledge exchange become more pertinent nowadays when new innovative laboratory technologies are used outside of Canada to aid the diagnosis of related AEs and HAEs. Evaluation of C1-INH function using contact-phase proteases as target, assessment of kinin formation during attacks through spontaneous kininogenase (amidase) activity, activation of kinin-forming zymogens, and HMWK plasma kininogen immunoblot and kinin catabolism have had a positive impact on the better understanding of pathophysiological mechanisms of BK-related AEs which serve as an umbrella for HAEs [8, 14, 38–40].

## Abbreviations

HAE: hereditary angioedema
BK: bradykinin
C1-INH-HAE: hereditary angioedema with C1 inhibitor deficiency
PK: prekallikrein
HMWK: high molecular weight kininogen
AAE: acquired angioedema
CHAEN: Canadian Hereditary Angioedema Network
ICL: In-Common Laboratories
nlC1-INH-HAE: hereditary angioedema with normal C1 inhibitor
KKS: Kallikrein–kinin system
